# Genetic Diversity of C_4_ Photosynthesis Pathway Genes in *Sorghum bicolor* (L.)

**DOI:** 10.3390/genes11070806

**Published:** 2020-07-16

**Authors:** Yongfu Tao, Barbara George-Jaeggli, Marie Bouteillé-Pallas, Shuaishuai Tai, Alan Cruickshank, David Jordan, Emma Mace

**Affiliations:** 1 Queensland Alliance for Agriculture and Food Innovation (QAAFI), The University of Queensland, Hermitage Research Facility, Warwick, QLD 4370, Australia; y.tao1@uq.edu.au (Y.T.); b.georgejaeggli@uq.edu.au (B.G.-J.); mariebouteille0384@gmail.com (M.B.-P.); david.jordan@uq.edu.au (D.J.); 2 Agri-Science Queensland, Department of Agriculture and Fisheries (DAF), Hermitage Research Facility, Warwick, QLD 4370, Australia; Alan.Cruickshank@daf.qld.gov.au; 3 BGI Genomics, BGI-Shenzhen, Shenzhen 518083, China; shuaishuai.tai@bgi.com

**Keywords:** sorghum, C_4_ pathway, genetic diversity, SNPs, domestication

## Abstract

C_4_ photosynthesis has evolved in over 60 different plant taxa and is an excellent example of convergent evolution. Plants using the C_4_ photosynthetic pathway have an efficiency advantage, particularly in hot and dry environments. They account for 23% of global primary production and include some of our most productive cereals. While previous genetic studies comparing phylogenetically related C_3_ and C_4_ species have elucidated the genetic diversity underpinning the C_4_ photosynthetic pathway, no previous studies have described the genetic diversity of the genes involved in this pathway within a C_4_ crop species. Enhanced understanding of the allelic diversity and selection signatures of genes in this pathway may present opportunities to improve photosynthetic efficiency, and ultimately yield, by exploiting natural variation. Here, we present the first genetic diversity survey of 8 known C_4_ gene families in an important C_4_ crop, *Sorghum bicolor* (L.) Moench, using sequence data of 48 genotypes covering wild and domesticated sorghum accessions. Average nucleotide diversity of C_4_ gene families varied more than 20-fold from the NADP-malate dehydrogenase (MDH) gene family (θπ = 0.2 × 10^−3^) to the pyruvate orthophosphate dikinase (PPDK) gene family (θπ = 5.21 × 10^−3^). Genetic diversity of C_4_ genes was reduced by 22.43% in cultivated sorghum compared to wild and weedy sorghum, indicating that the group of wild and weedy sorghum may constitute an untapped reservoir for alleles related to the C_4_ photosynthetic pathway. A SNP-level analysis identified purifying selection signals on C_4_ PPDK and carbonic anhydrase (CA) genes, and balancing selection signals on C_4_ PPDK-regulatory protein (RP) and phosphoenolpyruvate carboxylase (PEPC) genes. Allelic distribution of these C_4_ genes was consistent with selection signals detected. A better understanding of the genetic diversity of C4 pathway in sorghum paves the way for mining the natural allelic variation for the improvement of photosynthesis.

## 1. Introduction

C_4_ photosynthesis has independently evolved in more than 60 different plant taxa [1]. The main driver for this convergent evolution is the tendency of Ribulose-1,5-bisphosphate carboxylase (Rubisco), which catalyzes the net fixation of carbon dioxide (CO_2_) to also catalyze an unfavorable oxygenation reaction. This reaction produces toxic phosphoglycolate which has to be converted to useful metabolites requiring substantial metabolic energy [2,3]. This wasteful use of CO_2_ is termed photorespiration. Photorespiration becomes a major constraint of photosynthesis in situations where CO_2_ to O_2_ ratios are low and temperatures are high. The evolution of C_4_ photosynthesis coincided with declining atmospheric CO_2_ concentrations [1,4] as a mechanism to avoid photorespiration by concentrating CO_2_ in the vicinity of ribulose-1,5-bisphosphate carboxylase (Rubisco).

In the majority of C_4_ plants, this is achieved via spatial separation of the initial CO_2_ fixation and the Calvin–Benson–Bassham cycle in two different cell types, most often mesophyll cells and bundle sheath cells [5]. CO_2_ concentration in C_4_ bundle sheath cells is up to 10-fold higher than that found in C_3_ mesophyll cells [6]. At higher temperatures, C_4_ photosynthesis is not only more efficient compared with C_3_ photosynthesis in terms of reducing energy losses from photorespiration, but due to the improved efficiency of this pathway, it renders plants more nitrogen- and water-use efficient [7,8]. C_4_ plants are more productive than C_3_ plants in areas with high light intensities, warm temperatures, and low rainfall, such as the sub-tropical and tropical areas around the globe.

Many of the major crops that originated from warm and dry regions of the world, such as maize, sorghum, millet, sugarcane, *miscanthus*, and switchgrass, use the C_4_ pathway [9]. C_4_ crops account for an estimated 23% of global primary production [10]. Improved photosynthetic capacity has been suggested as the next frontier in lifting crop productivity [11]. The C_4_ photosynthesis pathway is a good starting point to improve photosynthetic capacity and resource efficiency in crop plants. Attempts are currently being undertaken to integrate characteristics of the C_4_ pathway into C_3_ crops [7,12,13,14].

However, possibly due to multiple independent evolutions of C_4_ photosynthesis in different plant taxa [1], large variation also exists among C_4_ species in terms of the biochemical pathway. It has long been known that three major biochemical subtypes—nicotinamide adenine dinucleotide phosphate-malic enzyme (NADP-ME), nicotinamide adenine dinucleotide-malic enzyme (NAD-ME) and phospho-enol-pyruvate carboxykinase (PCK)—exist among C_4_ species [15]. More recently, it has been suggested that mixtures among them exist [16] and that the subtypes vary in their performance under different environmental conditions, e.g., low light [17]. Especially among the grasses, which all of the C_4_ cereals belong to, differences in pathway and performance are likely to exist, as C_4_ photosynthesis has evolved at least 25 times in this group of plants [18]. Exploring such variation may provide avenues to further improve C_4_ photosynthetic efficiency [9].

Sorghum is an NADP-ME subtype C_4_ crop well-known for its adaption to drought and high temperatures. It provides staple food for over 500 million people in the semi-arid tropics of Africa and Asia; in addition to being an important source of feed, fiber, and biofuel. Due to these characteristics, it is expected to play an increasingly important role in meeting the challenges of feeding the world’s growing population under the threat of global warming. Substantial variation in photosynthesis and related traits has been revealed in sorghum [19,20,21,22,23], indicating existence of genetic variation of underlying genes. However, this variation has not yet been studied.

The recent assembly of whole-genome sequences for a wide range of wild and cultivated sorghum species [24,25,26] provides an excellent opportunity to explore genetic diversity of genes related to the C_4_ photosynthetic pathway. Several high-throughput comparative transcriptomics and evolutionary studies using C_3_ and C_4_ phylogenetically related species and cell-specific gene expression have elucidated the key genes and regulatory networks that underpin the C_4_ photosynthetic pathway [5,27,28,29,30,31,32,33,34,35,36,37]. In the present study, we explored the genetic variation in genes that have previously been identified as core C_4_ genes, mined their allelic diversity and investigated signatures of selection during domestication in sorghum.

## 2. Materials and Methods

### Identification of C_4_ Gene Families

**Figure 1:**
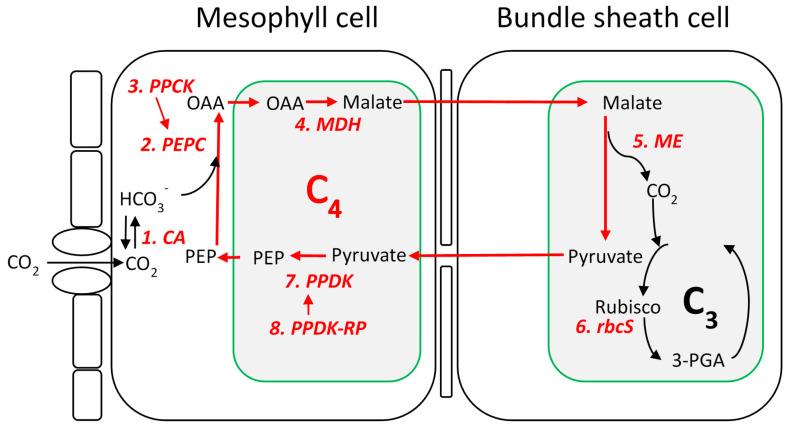
Diagram of the nicotinamide adenine dinucleotide phosphate-malic enzyme (NADP-ME) biosynthetic pathway of C_4_ photosynthesis (adapted from [40]). In the mesophyll cells, CO_2_ is converted to HCO_3_^−^ catalyzed by carbonic anhydrase (CA) and fixed into the four-carbon acid, oxaloacetate (OAA), by phosphoenolpyruvate carboxylase (PEPC). Phosphorylation of PEPC is carried out by PEPC kinase (PPCK). The OAA generated by PEPC is then reduced to malate by the NADP-malate dehydrogenase (NADP-MDH) or trans-aminated to aspartate. The resultant C_4_ acids, malate and aspartate, are transported to the bundle sheath and then decarboxylated in the vicinity of Rubisco to release CO_2_ and pyruvate. Pyruvate is transported back to mesophyll cells to regenerate PEP by pyruvate orthophosphate dikinase (PPDK), while CO_2_ enters the Calvin–Benson–Bassham cycle and is fixed by ribulose-1,5-bisphosphate carboxylase (Rubisco). Activation and inactivation of PPDK is catalyzed by PPDK regulatory protein (PPDK-RP).

This study focuses on 8 key proteins in the NADP-ME photosynthetic pathway in sorghum (Figure 1). A total of 9 genes encoding these proteins with expression and evolutionary evidence supporting their involvement in NADP-ME pathway (hereafter, referred as C_4_ genes), and their non-C_4_ isoforms in sorghum were extracted from two previous studies [38,39] (Table 1). These non-C_4_ isoforms are homologous of C_4_ genes but there was no evidences supporting their involvement in the NADP-ME photosynthetic pathway. Homology between these sorghum C_4_ genes and their non-C_4_ isoforms was further verified via a local blast strategy. Protein sequences of these 9 core C_4_ genes were extracted from the sorghum reference genome V3.1 and were blasted against the reference genome. Blast hits of each gene were filtered using the criteria: E-value <−10, sequence identity >60%, and alignment length >80%. All hits of the same gene satisfying the criteria were plotted based on –log (E-value); only hits of top –log (E-value) class were considered if clear differentiation among them was visualized, otherwise all hits were used.

## 3. Plant Material and Genomic Data

Sequence data of the identified C_4_ genes were extracted from 48 accessions of *Sorghum bicolor* with high mapping depth (~22× per accession, ranging from 16 to 45×) reported in previous studies [24,25,26]. These 48 accessions represent all major cultivated sorghum races and some wild progenitors (Table S1).

## 4. Gene-Level Population Genetic Analyses

Population genetic parameters including nucleotide diversity (θπ) [41], Tajima’s D [42], and Watterson’s Estimator (hW) [43] were directly calculated for each of the 27 genes using the Bio::PopGen::Statistics module. F_ST_ [44], which measures population differentiation, was also calculated for each of the 27 genes using the Bio::PopGen::PopStats module [26]. The Bio::PopGen::IO module was used to read input file, which was prepared using an in-house Perl script for calculation of these population genetic parameters.

The criteria used in Mace et al. (2013) were employed to identify genes under purifying selection and balancing selection, respectively. Criteria for purifying selection included: (1) θπ and hW < 5% of the empirical distribution in the cultivated group, (2) F_ST_ between the group of cultivated sorghum and the group of wild and weedy sorghum > 95% of the population pairwise distribution, (3) Tajima’s D < 0. Criteria for balancing selection included: (1) θπ and hW > 25% of the empirical distribution in the cultivated group, (2) F_ST_ between the group of cultivated sorghum and the group of wild and weedy sorghum < 90% of the population pairwise distribution, (3) Tajima’s D > 5% of the empirical distribution.

## 5. SNP-Level Identification of Selection Signature

Population genetics parameters including θπ, Tajima’s D, and F_ST_ between the group of cultivated sorghum and the group of wild and weedy sorghum were computed for these 27 genes using CDS sequence in PopGenome, a population genomics package implemented in the R environment (http://cran.r-project.org/) [45]. Specifically, commands diversity.stats, F_ST.stats, and neutrality.stats were called to calculate θπ, F_ST_, and Tajima’s D for each single nucleotide polymorphism (SNP), respectively, with a slide window of 1-bp and 1-bp step size. Functional annotation of each SNP was conducted using get.codons command. Fold decrease of θπ in the cultivated sorghum group compared to the group of wild and weedy sorghum was calculated to represent reduction of diversity (RoD). The following criteria were adopted to identify sites with signature of purifying selection: (1) A RoD greater than the average of neutral genes; (2) F_ST_ > 0; (3) Tajima’s D < 0. The following criteria were adopted to identify sites with signature of balancing selection: (1) An increase in diversity (IoD) in the cultivated group and the group of wild and weedy comparison; (2) F_ST_ > 0; (3) Tajima’s D > 0.

## 6. Phylogenetic and Haplotype Analysis

A phylogenetic tree was constructed based on CDS of all 27 genes from C_4_ gene families using the neighbor-joining method with default settings (bootstrapped 100 times; support threshold, 50%) in Geneious 8.1.2 (https://www.geneious.com/, Biomatters Ltd., Auckland, New Zealand). Analysis of haplotype network was conducted using a combination of the R package ape [46] and pegas [47]. All 48 sorghum accessions were classified into four groups: Cultivated, wild and weedy, *Guinea margaritiferum* and *S. propinquum* (Table S2).

## 7. Results

### Nucleotide Diversity of Core C_4_ Gene Families in Sorghum

**Figure 2:**
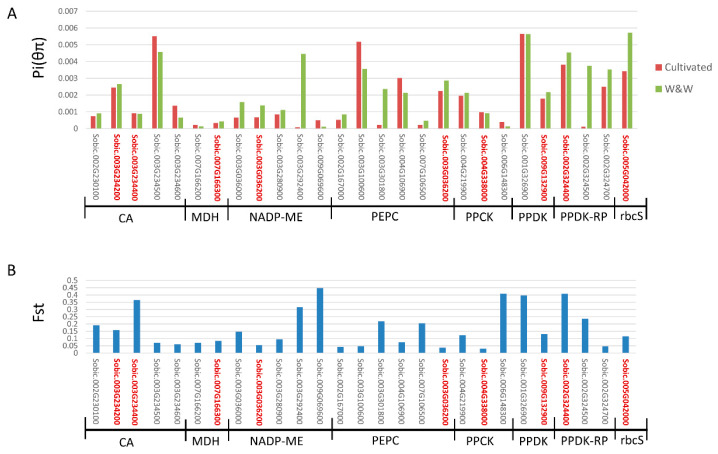
Genetic diversity and fixation index (F_ST_) of C_4_ gene families between cultivated sorghum and the wild and weedy group. (**A**) Genetic diversity (pi) for each of the C_4_ gene families. Gene IDs in red indicate core C_4_ genes. Red bars represent the pi of cultivated sorghum, while dark blue bars represent the pi of wild and weedy. (**B**) F_ST_ between cultivated and wild and weedy of each of C_4_ gene families. Gene IDs in red indicate core C_4_ genes.

Based on 9 genes corresponding to 8 core C_4_ enzymes in sorghum, 18 homologous genes were identified across the sorghum genome. In total, 5 CA genes, 2 NADP-MDH genes, 5 NADP-ME genes, 6 PEPC genes, 3 PPCK genes, 2 PPDK genes, 3 PPDK-RP genes, and 1 rbcS gene were identified (Table 1). Nucleotide diversity (θπ) of these 27 genes was investigated using sequence data of 48 genotypes covering wild and weedy, and cultivated sorghum (Mace et al., 2013). A total number of 4183 single nucleotide polymorphisms (SNPs) were identified in these 27 genes with 521 SNPs located in coding sequence (CDS) regions (Table 1). These C_4_ gene families displayed an average overall nucleotide diversity of θπ = 2.09 × 10^−3^, which is comparable to that of 130 housekeeping genes (θπ = 1.97 × 10^−3^, Mace et al., 2013) (*t*-test, *p*-value > 0.05). Nucleotide diversity varied dramatically among the C_4_ gene families, with the NADP-MDH genes displaying the lowest levels of diversity across all genotypes (average θπ = 0.25 × 10^−3^), followed by NADP-ME genes (θπ = 0.93 × 10^−3^), PPCK genes (θπ = 1.20 × 10^−3^), PEPC genes (θπ = 2.11 × 10^−3^), CA (θπ = 2.26 × 10^−3^), and PPDK-RP (θπ = 2.96 × 10^−3^), while PPDK genes showed the highest level of diversity (θπ = 5.21 × 10^−3^) (Table 2, Figure 2A). The only gene encoding ribulose bisphosphate carboxylase/oxygenase small-subunit (rbcS), Sobic.005G042000, had relatively high genetic diversity among C_4_ gene families with θπ = 4.32 × 10^−3^ across all 48 genotypes, 5.72 × 10^−3^ in the wild and weedy group, and 3.03 × 10^−3^ in the cultivated group.

Mixed trends were found when comparing C_4_ genes with non-C_4_ isoforms in each gene family with the average overall genetic diversity of C_4_ genes being comparable to that of their non-C_4_ counterpart (Table 2). The C_4_ PPDK-RP gene (Sobic.007G166300) and C_4_ NADP-MDH gene (Sobic.002G324400) had an overall θπ which was 161.76% and 79.85% higher than their non-C_4_ isoforms, respectively, whereas the θπ of the C_4_ PPDK gene (Sobic.009G132900) was 75.16% lower than that of the non-C_4_ PPDK isoform. Nucleotide diversity of C_4_ genes in the other gene families was within the range of variation of their non-C_4_ isoforms.

Genetic diversity across C_4_ gene families was significantly reduced during sorghum domestication (paired *t*-test, *p*-value < 0.05). Averaged across all C_4_ gene families genetic diversity was reduced by 22.44% in the domesticated compared with the wild and weedy group and when just the 9 core C_4_ genes were considered, the reduction was 22.98%. However, the reduction of genetic diversity during domestication in C_4_ genes was not significantly different from that in housekeeping genes (Table S2) (*t*-test, *p*-value > 0.05). Among the 27 genes, Sobic.003G292400, a non-C_4_ NADP-ME isoform, exhibited the most severe reduction in genetic diversity, with a reduction of 98.23%. The C_4_ version of that gene, the NADP-ME gene (Sobic.003G036200), showed the greatest loss of genetic diversity (51.89%) among the C_4_ genes, with an F_ST_ between the cultivated and wild and weedy groups of 0.06 (Figure 2B). In contrast, another non-C_4_ isoform of NADP-ME (Sobic.009G069600), a non-C_4_ isoform of PPCK (Sobic.006G148300), and a non-C_4_ CA isoform (Sobic.003G234600) showed a more than 2-fold increase in genetic diversity in the cultivated group.

## 8. Identification of Selection Signals during Domestication across the 27 Genes

The selection signature of these C_4_ gene families was firstly investigated at the gene level. Based on thresholds of genome-wide rankings described in Mace et al. (2013), only one gene (Sobic.001G326900, non-C_4_ PPDK isoform) was identified as being under balancing selection, which maintains diversity of selected genes, during sorghum domestication, while no gene was identified as being under purifying selection, which reduces diversity of selected genes (Table 1). Subsequent to this, a higher resolution detection of selection signature was conducted at the SNP level using the CDS of the 27 genes. Among 521 SNPs across 27 CDS, 176 were non-synonymous. The number of non-synonymous SNPs within genes varied from 19 in the non-C_4_ PPDK-RP isoform (Sobic.002G324700) to 0 in the C_4_ PPDK (Sobic.009G132900). The C_4_ PEPC gene (Sobic.010G160700) had the highest number of non-synonymous SNPs (9) among the 9 C_4_ genes (Table 1). In contrast to the gene-level analysis, SNP-level analysis identified 24 SNPs across 8 genes under purifying selection, including 7 non-synonymous SNPs in 6 genes (Table S3). Genes with SNPs under purifying selection included two C_4_ isoforms, PPDK (Sobic.009G132900) and CA (Sobic.003G234200), three of 4 non-C_4_ NADP-ME (Sobic.003G280900, Sobic.003G292400, Sobic.009G069600), both two non-C_4_ PPDK-RP (Sobic.002G324500, Sobic.002G324700), and a non-C_4_ PEPC gene (Sobic.007G106500). Among the 2 C_4_ genes with SNPs under selection, Sobic.009G132900 had 3 synonymous SNPs under purifying selection, while Sobic.003G234200 had a non-synonymous SNP under purifying selection.

A total of 60 SNPs across 8 genes were identified as being under balancing selection, 7 of which were non-synonymous SNPs distributed across 2 genes (Table S4). The non-C_4_ PPDK (Sobic.001G326900) had 24 SNPs under balancing selection including 5 non-synonymous SNPs, and additionally had an overall gene-level signature of balancing selection based on the previous analysis. Two C_4_ isoforms, PPDK-RP (Sobic.002G324400) and PEPC (Sobic.010G160700), were identified with 3 and 2 SNPs under balancing selection, respectively, although none of them were non-synonymous SNPs. Two non-C_4_ PEPC (Sobic.003G100600, Sobic.004G106900) were identified with SNPs under balancing selection, with Sobic.003G100600 having 21 SNPs including 2 non-synonymous SNPs exhibiting signatures of balancing selection. The other 2 genes with SNPs under balancing selection were a non-C_4_ CA isoform, Sobic.002G230100, and a non-C_4_ PPCK isoform, Sobic.004G219900.

## 9. Allelic Variation of Core C_4_ Genes under Selection in Sorghum

**Figure 3:**
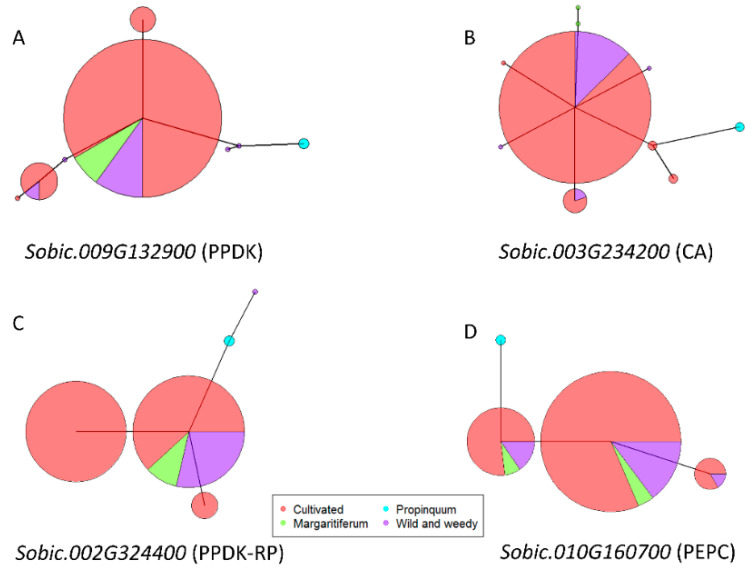
Haplotype network of 4 core C_4_ gene with selection signal based on individual SNP analysis. (**A**) The PPDK gene (Sobic.009G132900) with signal of purifying selection; (**B**) one of the CA genes (Sobic.003G234200) with signal of purifying selection; (**C**) the PPDK-RP gene (Sobic.002G324400) with signal of balancing selection; (**D**) the PEPC gene (Sobic.010G160700) with signal of balancing selection. Group classification of sorghum accessions used as detailed in Table S1. Color-coding as follows; cultivated sorghum (red), wild and weedy genotypes (purple), *Sorghum propinquum* (blue), and *Sorghum guinea margaritiferum* (green). The size of the circles in the haplotype networks is proportionate to the number of accessions with that haplotype. The branch length represents the genetic distance between two haplotypes.

A phylogenetic tree was constructed using the CDS of these 27 genes to depict the genetic relationship of 48 accessions (Figure S1). The inter-and intra-species distribution of private haplotypes of each gene is detailed in Table S5, with the majority (~90%) of the genes with private inter-species haplotypes from *S. propinquum*, e.g., 4 unique haplotypes were observed for the C_4_ isoform of PEPC, with the 2 *S. propinquum* accessions sharing a single private haplotype. To investigate allelic variation of 4 core C_4_ genes with SNPs under selection in sorghum, haplotype networks were constructed using CDS SNPs. Based on 16 SNPs within the CDS of the PPDK gene (Sobic.009G132900), 8 haplotypes were identified. Five haplotypes were identified in the wild and weedy genotypes, with 3 being private haplotypes and two of them being maintained in cultivated sorghum; two new haplotypes arose in cultivated sorghum after domestication (Figure 3A). Ten haplotypes of one CA gene (Sobic.003G234200) were revealed using 33 SNPs, with 4 distinct haplotypes being characterized by the wild and weedy genotypes. Two of the wild and weedy haplotypes were maintained in cultivated sorghum during domestication, with three new haplotypes arising after domestication (Figure 3B). The loss of wild and weedy haplotypes in cultivated sorghum in these two genes was consistent with the finding that they were under purifying selection.

The PPDK-RP gene (Sobic.002G324400) had 22 SNPs in the CDS, based on which 5 haplotypes were identified. Two haplotypes were characterized by the wild and weedy genotypes, with the main wild haplotype maintained and further diversifying into two new haplotypes in the cultivated group (Figure 3C). Based on 28 SNPs in the CDS of the C_4_ PEPC gene (Sobic.010G160700), 4 haplotypes were identified. Wild and weedy genotypes encompassed 3 haplotypes and all of them were maintained in cultivated sorghum (Figure 3D). *S. propinquum* had unique haplotypes across all 4 genes, while the *Sorghum bicolor* race *guinea margaritiferum* shared haplotypes with the wild and weedy genotypes in most cases, indicating a closer relationship with the wild and weedy group.

## 10. Discussion

The evolution of C_4_ photosynthesis has been studied extensively at the cross-species level with signals of adaptive evolution identified on key genes in the C_4_ pathway [28,34,48,49,50]. As the evolution of C_4_ photosynthesis is driven by environments characterized by low CO_2_ availability, such as hot and dry environments in which CO_2_ uptake is limited by stomatal closure, it is likely that within-species adaptive variation also exists. However, to our knowledge, studies of within-species allele diversity and signatures of selection on key genes in the C_4_ pathway have not previously been undertaken.

Knowledge of existing natural variation and levels of genetic diversity is a pre-requisite for the optimization of C_4_ photosynthesis. In this study, we performed the first investigation of the genetic diversity of C_4_ gene families within a C_4_ species using a collection of 48 sorghum lines. We focused on 9 C_4_ genes due to their reported key roles in C_4_ photosynthesis. Our collection of sorghum represents all major cultivated sorghum races, landraces, and wild progenitors, and captures a good proportion of genetic diversity within sorghum. Substantial variation of nucleotide diversity was observed among these 8 C_4_ gene families in sorghum, with the NADP-MDH gene family showing the least diversity and the PPDK gene family showing the greatest diversity. Nine core C_4_ genes also exhibited varying degrees of genetic diversity, ranging from θπ values of 5.04 × 10^−3^ and 4.32 × 10^−3^ in PPDK-RP and rbcS to θπ values of 0.33 × 10^−3^ and 0.67 × 10^−3^ in NADP-MDH and NADP-ME. However, despite such low levels of diversity, non-synonymous SNPs were identified in both NADP-MDH and NADP-ME (Table 1). C_4_ PPDK was the only gene which did not contain a non-synonymous SNP, despite its fairly large size (gene size, 12748bp; CDS, 2847bp), indicating the function of this gene is highly conserved.

Cultivated sorghum was domesticated more than five thousand years ago in Africa [51,52,53]. This artificial selection process has morphologically and physiologically reshaped sorghum to better suit human needs, and also resulted in substantial reduction of genetic diversity genome wide in cultivated sorghum compared with wild and weedy types [26,54,55]. In this study, reduction of genetic diversity during sorghum domestication was also observed in the C_4_ gene families, indicating that wild sorghum, as a repository for genetic diversity, might harbor alleles useful for improving C_4_ photosynthesis.

However, the overall reduction in diversity of C_4_ gene families was not significantly different from the genome-wide average, indicating that this gene family has not been under particularly strong selection pressure. Similarly, none of the 9 core C_4_ genes showed a domestication signal at the gene level. The absence of large sequence variation at the gene level is also consistent with previous evolutionary studies suggesting that relatively minor changes to pre-existing regulatory networks and the use of pre-existing cis-elements were often sufficient to recruit genes into the C_4_ pathway [56,57]. The C_4_ isoform of the NADP-ME gene found in maize and sorghum is one such gene that has been found to be activated for C_4_ photosynthesis via subtle changes to its promoter, while the rest of the gene is highly conserved [33]. This is consistent with the low diversity in this gene family observed in our study.

A further high-resolution investigation of domestication signature at the SNP level revealed 2 C_4_ genes, PPDK (Sobic.009G132900) and CA (Sobic.003G234200), with SNPs under purifying selection, while the other 2 C_4_ genes, PPDK-RP (Sobic.002G324400) and PEPC (Sobic.010G160700), were identified with SNPs under balancing selection. Previous studies have demonstrated that SNP-level analysis using less stringent criteria is superior for capturing soft selection signals compared with genome-wide ranking [54,58]. However, the higher sensitivity may come with a cost of a greater chance of false positives, and therefore requires cautious interpretation. The contrasting selection signals on genes from the same pathway within taxa found in this study was also reported previously in signal transduction pathways [59] and the starch biosynthesis pathway [60].

The C_4_ isoforms of PPDK and PEPC were also found to show signals of positive selection in a previous cross-species evolutionary study using orthologous groups from closely related C_3_ and C_4_ grass species including sorghum [28]. PPDK and PPDK-RP regulate the regeneration of PEP and as such have a direct effect on CO_2_ assimilation rate [61], especially under cool temperatures [62,63]. However, it is thought that only minor changes to the enzyme properties of PPDK were sufficient to recruit it into the C_4_ pathway and its residues and regions involved in catalyzes are highly conserved in C_4_ species [64], possibly validating the fact that only soft selection signals via SNP-level were found for the C_4_ isoform of the PPDK gene in our study.

PEPC is also regarded as a potential limiting step in the assimilation of CO_2_, and variation of its affinity for CO_2_/HCO_3_^−^ amongst species has been documented [65,66,67]. CA is also critical to C_4_ photosynthesis as it catalyzes the first step of the C_4_ pathway, converting CO_2_ to HCO_3_^−^ [68]. It was reported in the C_4_ dicot *Flaveria bidentis*, where antisense plants with <10% of wild-type CA activity required high CO_2_ for growth and showed reduced CO_2_ assimilation rates [69,70]. Recent experiments showed CA and PEPC will be more limiting when stomates are partially closed, e.g., under water limitation [71].

The signal of soft purifying selection on PPDK and CA may suggest the C_4_ pathway was indirectly improved during sorghum domestication. Without photosynthetic rate being a direct selection target in breeding programs, a steady increase in leaf photosynthetic rate over time of cultivar release has been shown in other cereals, e.g., in Australian bread wheat [72]. The balancing selection signal on C_4_ PPDK-RP and PEPC may reflect adaptation to diverse environments, as both PPDK-RP and PEPC are associated with abiotic stress [73,74]. Interestingly, within the PPDK-RP and PPDK gene families, the non-C_4_ genes all showed selection signals contrasting with their C_4_ counterparts with both two non-C_4_ PPDK-RP (Sobic.002G324500, Sobic.002G324700) containing SNPs under purifying selection and the non-C_4_ PPDK (Sobic.001G326900) containing SNPs under balancing selection.

After domestication, sorghum was introduced from tropical to temperate areas, and adapted to divergent local environments. New mutations also arose during this diversification process, and played an important role in local adaptation. In the haplotype analysis, these haplotypes unique to cultivated sorghum are likely to be young alleles arising after domestication, while haplotypes unique to the wild progenitor indicate that some haplotypes were lost during domestication of sorghum. Nevertheless, the loss of wild haplotypes of C_4_ genes in cultivated sorghum does not mean these haplotypes are inferior in terms of photosynthetic efficiency, as photosynthesis was not specifically targeted during sorghum domestication [11]. On the contrary, bringing these wild haplotypes back to breeding programs after evaluation of their functions may enrich breeders’ toolkits to manipulate photosynthetic efficiency, ultimately contributing to yield improvements.

C_4_ photosynthesis has been well studied over the past 50 years and key components of this complex pathway have been identified following the advent of transgenic and sequencing technologies [9]. Understanding the genetic diversity of the key enzymes of the C_4_ pathway is an important step towards mining the natural allelic variation for the improvement of photosynthesis. 

Further investigation of these allelic variation to link them with agronomical traits will provide new targets for sorghum improvement [75].

## Figures and Tables

**Table 1:** Single nucleotide polymorphism (SNP) information and selection signals across 27 genes from C_4_ gene families.

Gene ID	Enzyme	GL	CDSL	NoS	NoSiC	NoNS	NoSS	UPSGL	UBSGL	NoSUPS	NoNSUPS	NoSUBS	NoNSUBS
Sobic.002G230100	CA	4823	1014	115	14	4	10	No	No	0	0	1	0
**Sobic.003G234200**	CA	10440	1371	475	33	7	26	No	No	1	1	0	0
**Sobic.003G234400**	CA	4749	615	138	13	3	10	No	No	0	0	0	0
Sobic.003G234500	CA	2986	609	173	11	5	6	No	No	0	0	0	0
Sobic.003G234600	CA	4750	771	210	18	10	8	No	No	0	0	0	0
Sobic.007G166200	NADP-MDH	3354	1308	53	11	6	5	No	No	0	0	0	0
**Sobic.007G166300**	NADP-MDH	3816	1290	108	12	3	9	No	No	0	0	0	0
Sobic.003G036000	NADP-ME	6107	1941	111	11	4	7	No	No	0	0	0	0
**Sobic.003G036200**	NADP-ME	5447	1911	141	12	3	9	No	No	0	0	0	0
Sobic.003G280900	NADP-ME	5691	1782	175	22	13	9	No	No	1	1	0	0
Sobic.003G292400	NADP-ME	4527	1782	95	22	8	14	No	No	10	2	0	0
Sobic.009G069600	NADP-ME	3624	1713	118	34	10	24	No	No	3	1	0	0
Sobic.002G167000	PEPC	5632	2904	41	11	6	5	No	No	0	0	0	0
Sobic.003G100600	PEPC	8881	3117	371	43	9	34	No	No	0	0	21	2
Sobic.003G301800	PEPC	7610	2901	138	19	3	17	No	No	0	0	0	0
Sobic.004G106900	PEPC	6977	2883	146	34	5	29	No	No	0	0	7	0
Sobic.007G106500	PEPC	5616	2895	64	12	8	4	No	No	1	1	0	0
**Sobic.010G160700**	PEPC	6647	3087	193	28	9	19	No	No	0	0	2	0
Sobic.004G219900	PPCK	1612	924	40	9	1	8	No	No	0	0	2	0
**Sobic.004G338000**	PPCK	1749	855	37	9	4	4	No	No	0	0	0	0
Sobic.006G148300	PPCK	1997	900	64	4	1	3	No	No	0	0	0	0
Sobic.001G326900	PPDK	8494	2730	321	46	18	28	No	Yes	0	0	24	5
**Sobic.009G132900**	PPDK	12748	2847	441	16	0	16	No	No	3	0	0	0
**Sobic.002G324400**	PPDK-RP	2507	1290	79	22	8	14	No	No	0	0	3	0
Sobic.002G324500	PPDK-RP	3072	1260	69	20	5	15	No	No	4	0	0	0
Sobic.002G324700	PPDK-RP	4662	1587	222	28	19	9	No	No	1	1	2	2
**Sobic.005G042000**	RbcS	1556	510	45	7	4	3	No	No	0	0	0	0

Gene ID is according to sorghum reference genome V3.1. Gene IDs in bold indicate their C_4_ genes. Enzyme: Encoded enzyme. GL: Gene length. CDSL: Length of coding sequence (CDS). NoS: Total number of SNPs identified across the gene. NoSiC: Number of SNPs identified in CDS. NoNS: Number of non-synonymous SNPs. NoSS: Number of synonymous SNPs. UPSGL: Under purifying selection based on gene level analysis. UBSGL: Under balancing selection based on gene level analysis. NoSUPS: Number of SNPs under purifying selection. NoNSUPS: Number of non-synonymous SNPs under purifying selection. NoSUBS: Number of SNPs under balancing selection. NoNSUBS: Number of non-synonymous SNPs under balancing selection.

**Table 2:** Genetic diversity (θπ) and fixation index (F_ST_) of 27 genes from C_4_ gene families.

GeneID	Enzyme	θπ–All	θπ-Cultivated	θπ-W&W	F_ST_
Sobic.002G230100	CA	0.80	0.74	0.90	0.19
**Sobic.003G234200**	CA	2.65	2.46	2.66	0.16
**Sobic.003G234400**	CA	1.01	0.91	0.88	0.37
Sobic.003G234500	CA	5.55	5.51	4.56	0.07
Sobic.003G234600	CA	1.27	1.35	0.65	0.06
Sobic.007G166200	NADP-MDH	0.18	0.21	0.13	0.07
**Sobic.007G166300**	NADP-MDH	0.33	0.33	0.42	0.08
Sobic.003G036000	NADP-ME	0.88	0.65	1.59	0.15
**Sobic.003G036200**	NADP-ME	0.89	0.67	1.39	0.06
Sobic.003G280900	NADP-ME	0.93	0.85	1.11	0.09
Sobic.003G292400	NADP-ME	1.43	0.08	4.44	0.32
Sobic.009G069600	NADP-ME	0.52	0.49	0.10	0.45
Sobic.002G167000	PEPC	0.58	0.51	0.85	0.04
Sobic.003G100600	PEPC	5.36	5.18	3.56	0.05
Sobic.003G301800	PEPC	0.64	0.22	2.37	0.22
Sobic.004G106900	PEPC	3.18	3.02	2.14	0.07
Sobic.007G106500	PEPC	0.44	0.22	0.47	0.21
**Sobic.010G160700**	PEPC	2.49	2.25	2.86	0.04
Sobic.004G219900	PPCK	2.08	1.94	2.12	0.12
**Sobic.004G338000**	PPCK	1.03	0.96	0.91	0.03
Sobic.006G148300	PPCK	0.48	0.39	0.13	0.41
Sobic.001G326900	PPDK	8.34	5.64	5.64	0.40
**Sobic.009G132900**	PPDK	2.07	1.79	2.19	0.13
**Sobic.002G324400**	PPDK-RP	5.04	3.82	4.55	0.41
Sobic.002G324500	PPDK-RP	1.27	0.10	3.75	0.24
Sobic.002G324700	PPDK-RP	2.58	2.50	3.51	0.05
**Sobic.005G042000**	rbcS	4.32	3.41	5.72	0.12

Gene ID is according to sorghum reference genome V3.1. Gene IDs in bold indicate the C4 gene versions. Enzyme: Encoded enzyme. θπ-All: Nucleotide diversity across all 48 genotypes. θπ-Cultivated: Nucleotide diversity across cultivated genotypes. θπ-W&W: Nucleotide diversity across wild and weedy genotypes. All θπ values are in unites of per kb. F_ST_: Fixation index between cultivated genotypes and wild and weedy genotypes.

## Supplementary Materials

The following are available online at https://www.mdpi.com/2073-4425/11/7/806/s1. Table S1: List of re-sequenced sorghum accessions and their racial and geographic origins. Table S2: List of housekeeping genes and their genetic diversity. Table S3 List of SNPs under purifying selection. Table S4: List of SNPs under balancing selection. Table S5: Inter- and intra-species distribution of private alleles across 27 genes from C_4_ gene families. Figure S1: Phylogenetic tree of 48 sorghum accessions based on CDS of 27 genes from C_4_ gene families.

## Abbreviations

CA, carbonic anhydrase; PEPC, phosphoenolpyruvate carboxylase; PPCK, phosphoenolpyruvate carboxylase kinase; NADP-MDH, NADP-malate dehydrogenase; NADP-ME, NADP-malic enzyme; PPDK, pyruvate orthophosphate dikinase; PPDK-RP, PPDK regulatory protein; RbcS, ribulose bisphosphate carboxylase/oxygenase small-subunit.
