# Supplementary figures and images for: Genetic Diversity of C4 Photosynthesis Pathway Genes in Sorghum bicolor (L.)
Source: Genes (Basel). 2020 Jul 16;11(7):806. doi: 10.3390/genes11070806 (PMC7397294; doi:10.3390/genes11070806)

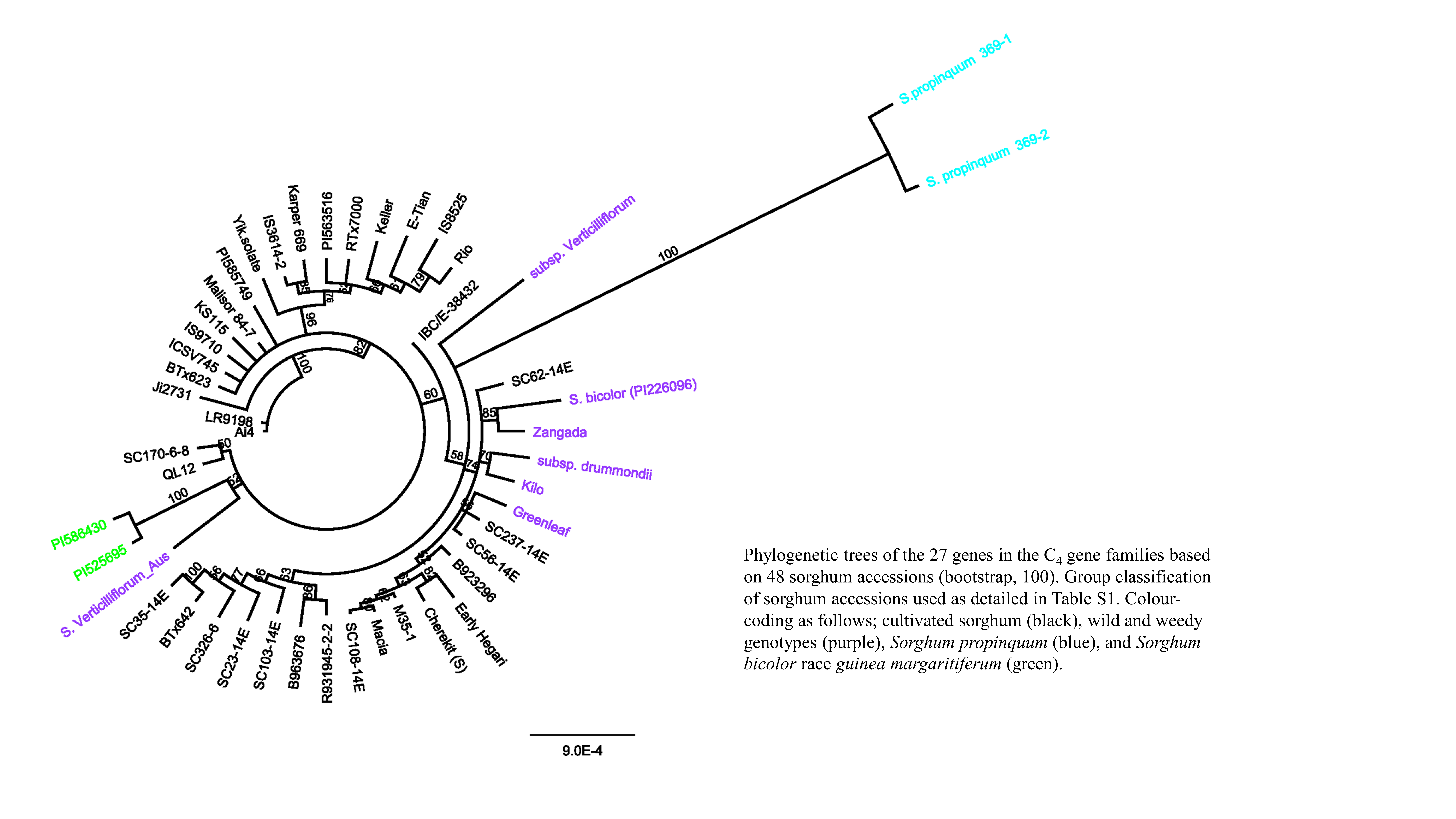

Supplement: Supplementary file 1 [file genes-11-00806-s001.zip › Supplementary files/Supp Fig1.tif]
